# Molecular characterization and lineage analysis of canine astrovirus strains from dogs with gastrointestinal disease in Ecuador based on ORF-2 gene analysis

**DOI:** 10.3389/fvets.2024.1505903

**Published:** 2025-02-03

**Authors:** Anthony Loor-Giler, Silvana Santander-Parra, Sara Castillo-Reyes, Martín Campos, Renan Mena-Perez, Santiago Prado-Chiriboga, Luis Nunez

**Affiliations:** ^1^Laboratorios de Investigación, Dirección General de Investigación, Universidad de las Américas (UDLA), Quito, Ecuador; ^2^Facultad de Ingeniería y Ciencias Aplicadas, Carrera de Ingeniería en Biotecnología, Universidad de Las Américas (UDLA), Quito, Ecuador; ^3^Facultad de Ciencias de la Salud, Carrera de Medicina Veterinaria, Universidad de Las Americas (UDLA), Quito, Ecuador; ^4^Facultad de Industrias Agropecuarias y Ciencias Ambientales, Carrera Agropecuaria, Universidad Politécnica Estatal del Carchi (UPEC), Tulcán, Ecuador; ^5^Facultad de Ciencias Veterinarias, Universidad Nacional de Rosario (UNR), Santa Fe, Argentina; ^6^Facultad de Medicina Veterinaria y Zootecnia, Universidad Central Del Ecuador, Quito, Ecuador; ^7^Clínica Veterinaria Docente, Universidad de Las Américas (UDLA), Quito, Ecuador; ^8^One Health Research Group, Facultad de Ciencias de la Salud, Universidad de Las Américas (UDLA), Quito, Ecuador

**Keywords:** CaAstV, gastroenteric disease, RT-qPCR, linages, protein structure

## Abstract

Canine Astrovirus (CaAstV) part of the *Astroviridae* family and genus *Mamastrovirus*, is a linear RNA virus with a genome of approximately 6.6 kb with three open reading frames (ORF): ORF1a and ORF1b, which code for the most conserved non-structural proteins, and ORF2, which code for the capsid protein, the most variable region of the genome. This pathogen has been linked to gastrointestinal infections, primarily causing symptoms such as vomiting, diarrhea, lethargy and severe dehydration, mainly in co-infection with other enteric viruses. In the present study, the presence of CaAstV was identified in dogs with gastrointestinal disease in Ecuador using RT-qPCR with hydrolysis probes, these samples have also tested positive for canine parvovirus type 2 (CPV-2) and canine coronavirus (CCoV). Positive samples were used for end-point RT-PCR amplification and sequencing of ORF-2 using Sanger technology. The sequences were subjected to phylogenetic analysis to determine lineages and possible recombination events. Of the 502 samples tested, 336 were found to be positive for CaAstV, 49.4% in co-infection with CPV-2, 1% in co-infection with CCoV, and 4% in simultaneous infection with all three viruses. The presence of 4 of the 5 previously reported CaAstV lineages were identified, and three possible recombinant strains were identified. Given the high frequency of CaAstV infections in dogs with gastroenteritis and its high genetic variability, it emphasizes the need to implement routine diagnostic measures that include this pathogen as one of the main causes of the disease and a risk agent in case of multiple infections.

## 1 Introduction

Astroviruses (AstV) are non-enveloped viruses of 28–30 nm in diameter, usually star-shaped, belonging to the *Astroviridae* family. They are classified as *Mamastroviruses* (MAstV) with at least 19 species infecting mammals and *Avastroviruses* (AvAstV) with three species infecting birds, according to the International Committee on Taxonomy of Viruses (ICTV) (1, 2). However, new types of AstV have recently been reported in both mammals and birds as emerging pathogens associated with intestinal diseases. AstV-like particles were first identified in puppies with diarrhea in the United States in 1980 by electron microscopy (EM). Following this, there were few reports of similar findings both with and without diarrhea, with a higher prevalence observed in dogs suffering from gastrointestinal infectious diseases (GID) (1, 3–6).

Canine Astrovirus (CaAstV), classified as MAstV 5, was first fully sequenced in 2015 (7), where a ~ 6.6 kb of viral genome with three Open Reading Frames (ORFs) was identified (5, 7). ORF1a at the 5′ end coding for viral protease, ORF1b coding for RNA-dependent RNA Polymerase (RdRP) and ORF2 at the 3′ end coding for capsid protein. Previous analyses of CaAstV genomes classify ORF2 as the most variable and antigenic region of the virus determining the identification of phylogenetically generated strains and lineages (8). Consequently, it has been proposed that this hypervariable region plays a fundamental role in the binding of the virus to target cells through interactions with cell receptors, and in the neutralization of antibodies, due to the presence of these hypervariable regions, as well as in heterologous immunity (9–11).

Recent research has shown that the CaAstV genome changes over time, often as a related to genetic recombination (9, 10). Cases of genetic recombination between CaAstV strains have been reported and cross-species transmission scenarios have been suggested, indicating risks of the emergence of new astroviruses with conserved pathogenic capabilities (6, 9). The infectious capacity of different lineages of the virus remains unclear compared to other *Mamastrovirus* species, which have been shown to cause enteric symptoms as well as affect the liver, kidneys, and, in certain instances, the nervous system, including to the brain (12, 13).

In canines, this virus is identified by causing vomiting, diarrhea, severe dehydration, depression and lack of appetite (6, 14, 15). However, due to the multifactorial nature of these symptoms, the diagnosis of this virus is requires specific techniques. PCR and qPCR have been the gold standard for the diagnosis of these viruses, and validated techniques have been developed for the identification of the pathogen (13, 14, 16). The presence of CaAstV has been identified in dogs with gastroenteritis in the United States, China, Japan, Italy, United Kingdom, Australia and recently in some Latin American countries; therefore, it is classified in canine clinics as an emerging pathogen (5, 7, 11, 17, 18).

In addition, CaAstV is commonly found in co-infection with other enteric viruses such as canine parvovirus (CPV), canine coronavirus (CCoV), canine distemper virus (CDV) (17, 19, 20), and studies are needed to determine the true pathogenic role of canine astrovirus in both single and co-infection conditions. Various studies have shown that CaAstVs have spread in the canine population and have a wide genetic diversity (9, 10, 19). This study aimed to identify the presence of CaAstV and to characterize it at a molecular level. It is necessary to evaluate the genetic variability of strains circulating within the country in comparison to previously documented canine samples of varying ages from animals presenting enteric issues across various cities in Ecuador.

## 2 Methods

### 2.1 Sampling

In the present study, 502 fecal samples from dogs of different ages and with symptoms of enteric disease, primarily diarrhea, vomiting, fever, loss of appetite, and dehydration, were processed. The samples were received at the veterinary clinic of the Universidad de Las Americas from different provinces of Ecuador: Carchi (65 samples); Guayas (58 samples); Imbabura (28 samples); Pichincha (286 samples); Santo Domingo de los Tsachilas (61 samples). In order to determine the causal agent of the symptoms, they were routinely tested for canine parvovirus type 2 (CPV-2) and canine coronavirus (CCoV; Supplementary Table 1), and, for the purposes of this study, they were analyzed to identify CaAstV. Therefore, these samples will be used to determine the presence of the virus and the molecular characterization of the current CaAstV present in Ecuador. All procedures conducted in the present investigation were performed in accordance with the guidelines and the approval of the Committee on the Care and Use of Laboratory and Domestic Animal resources of the Agency of Regulation and Control of Phytosanitary and Animal Health of Ecuador (AGROCALIDAD), under number #INT/DA/019.

### 2.2 RNA extraction

A suspension 1:1 of the sample was made in a 2 mL microcentrifuge microtube with 1 mL of 1X phosphate-buffered saline (PBS) pH 7.4. The sample was homogenized and frozen at −80°C for 10 min, then thawed in a water bath at 56°C for 1 min and again homogenized. This procedure was repeated three times, then the samples were centrifuged at 12,000× g for 30 min. A 250 μL aliquot part of the supernatant was placed in a 1.5 mL microcentrifuge microtube and subjected to RNA extraction using TRIzol Reagent (Invitrogen by Life Technologies, Carlsbad, CA, United States), according to the manufacturer’s instructions.

### 2.3 RT-qPCR assay for CaAstV detection

For CaAstV detection, a 2-step RT-qPCR was performed. For cDNA formation, 8 μL of RNA extracted from samples was subjected to reverse transcription using the SuperScript™ III Reverse Transcriptase (Invitrogen™ Van Allen Way, Carlsbad, California, United States), with 1 μL of oligo (dT)20 (50 μM) and 1 μM of random hexamer primer according to the conditions and concentrations recommended by the manufacturers.

In this study, 2 pairs of primers and a probe were used for the detection of CaAstV targeting the ORF2 gene (Table 1). The qPCR reaction was used with a final volume of 25 μL, 10 μL of TaqMan™ Universal Master Mix II (Applied Biosystems, Carlsbad CA 92008, United States), 0.2uM of each primer, 0.05 μM of the probe, 5 μL de cDNA, and UltraPure™ DNAse/RNAse-Free Distilled Water dH2O (Invitrogen by Thermofisher Scientific, NY 14072 United States). The amplification protocol was used according to the indications of the reagent in standard mode: 2 min cycle at 50°C for enzyme activation, 2 min cycle at 95°C for initial denaturation, and 40 cycles at 95°C for 3 s denaturation and 56°C for 30 s for annealing, and 72°C for extension of the DNA template.

**Table 1:** Primers used in this study.

Primer	Gene	Assay	Sequences (5′ – 3′)	Product	Reference
CAstV-QF	ORF2	RT-qPCR	CAGAGCAATGGTCAATGA	79 bp	(30)
CAstV-QR			CTCACTTAGTGTAGGGAGA		
CAstV-QP			CY5-CGCTCAGCCTGGTCCTCTGG-BHQ2		
CaAstV 5F		RT-PCR	GAAAGAACTTAGGGATGA	189 bp	(21)
CaAstV 5R			GCCTAAACTAATCTAACTTAACTAA		
CaAstV 4F			CDATYACCCARACTGCTACA	1973 bp	
CaAstV 4R			TTCATCCCTAAGTTCTTTCTCA		
CaAstV 3F			ATTCCACGCCTGCCTACC	1,379 bp	
CaAstV 3R			GATGTAGCAGTTTGGGTA		
CaAstV 3.2F			ATGTGGGTTAAGCCTGAAAAAGTCA	680 bp	This study
CaAstV 3.2R			AGTAGATGTAGCAGTTTGGGTGATT		
CaAstV Int-1		Sequencing	AGGCATGACTATGAACGCATCA	–	
CaAstV Int-2			TGCCATAACTTGTATGGGGC		

For the standard curve construction, a cDNA of CaAstV positive sample was subjected to endpoint PCR, for amplifying a ORF2 gene fragment using the primers CaAstV 4F and 4R (Table 1). The RT-PCR product was subjected to enzymatic purification using ExoSAP-IT Express (Applied Biosystems by Thermofisher Scientific, California, CA, United States) according to the manufacturer’s instructions. The purified amplicon was quantified using NANO Drop equipment (Thermo Fisher Scientific, Carlsbad, CA, United States). The DNA Copy Number and dilution Calculator web tool (Thermo Fisher Scientific) was used to calculate the quantity of recombinant DNA necessary to make the first dilution with a known quantity of DNA copies, then tenfold dilutions from 10^9^ copies to 10^0^ copies were prepared to determine the sensitivity and amplification efficiency of the RT-qPCR assay.

### 2.4 Sequencing of ORF-2 gene

Positive samples with more than 1,000 gene copies per μL were selected and subjected to an end-point RT-PCR protocol to amplify the entire ORF2 gene using the previously described protocol (21). The RT-PCR product was subjected to enzymatic purification using ExoSAP-IT Express (Applied Bio-systems by Thermofisher Scientific, California, CA, United States) according to the manufacturer’s instructions. The purified RT-PCR product was used for Sanger-type sequencing in the forward and reverse directions using a BigDye® Terminator v3.1 Cycle Sequencing kit (Thermo Fisher Scientific, Carlsbad, CA, United States), and sequencing reactions were performed with an ABI 3730 DNA Analyzer (Thermo Fisher Scientific, Carlsbad, CA, United States) using the primer walking strategy to obtain the complete sequence of the ORF2 gene (Table 1).

### 2.5 Phylogenetic analyses

**T**he electropherograms obtained were analyzed and edited using the Genious PRIME 2020.2.1 program (22); ORF finder tool was used to complement the sequencing runs and obtain the complete CDS of the ORF2 gene. The BLAST tool was also used to compare the similarity of each obtained sequence (Supplementary Table 1) with others of CaAstV present in the GenBank. A multiple sequence alignment was built with the ORF2 obtained sequences and other sequences of different strains of the CaAstV existing in the GenBank, both for the nucleotide sequence and in amino acids sequence using the Clustal X 2.1 software (23). Subsequently, a phylogenetic analysis was performed for amino acid sequences using the MEGA 7: Molecular Evolutionary Genetics Analysis version 7 (24), based on complete ORF2 coding protein, with a Neighbor-Joining statistical method created by a Jones-Taylor-Thornton (JTT) model using the boot-strap method with 1,000 replicates. The nucleotide and amino acid sequences were used to determine the variations among them and their similarity to existing variants in the GenBank database of CaAstV strains that have been reported from around the world.

### 2.6 Recombination analysis

The groups of nucleotides sequences that showed less than 85% similarity with the sequences collected from GenBank were selected for recombination event analysis. The nucleotide sequences that were previously identified as belonging to lineages 1 to 4 were grouped into lineage consensuses, taking into account that the variation between the sequences of their respective lineages is less than 2%. Furthermore, a consensus was generated for the hypothetical new lineage that was reported in Thailand in 2023 (10, 12). The sequences selected for analysis were aligned using Clustal X 2.1 software (23), along with the lineage consensuses made from the entire ORF2 gene and analyzed using RDP4 software version v4.43 (25), in order to determine recombination events using automated detection algorithm settings.

### 2.7 Capsid protein structure prediction

The amino acid sequences corresponding to the ORF2 of each lineage and of the samples considered possible recombinants were subjected to an in-silico protein folding prediction algorithm using AlphaFold-2 (26) and analyzed in Pymol (27). There is no information on a 3D protein model of the dog astrovirus capsid protein, so the protein models are approximations and should not be taken as definitive versions.

### 2.8 Statistical analysis

The data distribution was assessed using a Shapiro–Wilk test. An analysis of the samples examined was conducted based on the variables of age, locality, and the results obtained from RT-qPCR. A Chi-square test was performed to determine if there were statistically significant differences in the presence of CaAstV between canines of different ages and according to the locality in which the samples were collected using Jamovi 2.3.24.

## 3 Results

### 3.1 Detection of CaAstV by RT-qPCR

The calibration curve generated for the detection and quantification method for CaAstV by RT-qPCR with hydrolysis probes resulted in 97.5% efficiency. Additionally, the assay showed a limit of detection (LoD) and limit of quantification (LOQ) of up to one copy of viral genetic material. Of the 502 samples from dogs with gastroenteritis submitted to the method using absolute quantification, the presence of CaAstV was identified in 66.93% of them. The data showed a normal distribution according to the Shapiro-wilks test (*p* < 0.001).

#### 3.1.1 Distribution of CaAstV by age

When analyzing the presence of CaAstV in relation to the age of the canines (Table 2), it was observed that the group from 3 to 12 months of age presented the highest number of positive samples, with 148 positive samples corresponding to 44.05% of the total number of positive samples, showing significant differences with the other groups (*p*-value<0.005). On the other hand, the group of canines over 108 months of age presented the lowest number of positive samples, with only 2% of the total number resulted in positive samples for CaAstV. A low number of samples were obtained from dogs with gastroenteritis of dogs over 108 months. The 13–48 months group showed the highest number of negative samples of CaAstV in all groups (*n* = 67).

**Table 2:** CaAstV detection based on age groups in samples of canine patients.

CaAstV detection by RT-qPCR							
Age (months)							
CaAstV	0–2	3–12	13–48	54–96	108–120	132–180	Total
Negative	32(28.82%)	46(23.71%)	67(46.85%)	14(37.84%)	4(36.36%)	3(50%)	166
Positive	79(71.17%)	*148(76.29%)	76(53.15%)	23(62.16%)	7(63.64)	3(50%)	336
Total	111(22.11%)	194(38.65%)	143(28.49%)	37(7.37%)	11(2.19%)	6(1.20%)	502

*Statistic differences. Percentage of positives and negatives calculated based on the total group and percentage of the total calculated based on the total number of samples.

In each age group of canines tested, the average viral load was similar (Table 3), with the highest CaAstV viral load in the 13–48 months group and the lowest in the 54–96 months group.

**Table 3:** Quantification of CaAstV based on the RT-qPCR assay in age groups average and maximum of gene copies.

Quantification of CaAstV by RT-qPCR						
Age (Months)	0 to 2	3 to 12	13 to 48	54 to 96	108 to 120	132 to 180
GC Average	3.66×10^8	4.40× 10^8	3.23×10^9	5.7×10^6	2.20×10^9	1.60×10^7
GC Maximum	2.58×10^10	2.40×10^10	1.74×10^11	7.63×10^7	2.19×10^10	9.57×10^7

GC, Gene copies.

#### 3.1.2 Distribution of CaAstV by locality

All sampled provinces presented CaAstV positivity. Pichincha had the highest percentage of positive samples (36.85%), while Chimborazo had the lowest (0.80%), representing 65.84 and 100% of their respective sample groups (Table 4). However, Pichincha corresponds to the most sampled locality and Chimborazo to the least sampled site, so the data may be biased. The provinces of Guayas, Imbabura and Chimborazo presented the highest percentages of positivity with respect to the total number of samples obtained from each province, while Carchi presented the lowest percentage of positivity for CaAstV in the samples analyzed. There were no significant differences between the presence or absence of CaAstV in any province.

**Table 4:** Distribution of CaAstV samples in Ecuadorian provinces.

CaAstV detection by RT-qPCR							
Locality (province)							
CaAstV	Carchi	Chimborazo	Guayas	Imbabura	Pichincha	Santo Domingo de los Tsachillas	Total
Negative	44(73.33%)	0(0%)	2(3.45%)	3(10.71%)	96(34.16%)	21(29.58%)	166
Positive	16(26.67%)	4(100%)	56(96.55%)	25(89.29%)	185(65.84%)	50(70.42%)	336
Total	60(11.95%)	4(0.8%)	58(11.55%)	28(5.58%)	281(55.98%)	71(14.14%)	502

Percentage of positives and negatives samples calculated based on the total group and percentage of the total calculation based on the total number of samples.

#### 3.1.3 Co-infection analysis

CaAstV was found as a single infection in only 12.5% of the samples tested, lower than single infections found with CPV-2 in 24.3% but higher than single infections found with CCoV in 0.4% (Table 5). On the other hand, co-infection was found more frequently with CPV-2 in 73.8% of positive samples for CaAstV and considerably less frequently with CCoV in 1.5%. In the samples analyzed, 4% showed co-infection of the three viruses in this study.

**Table 5:** Combinations of positive samples for CaAstV, CPV-2 and CCoV.

Combination	CaAstV	CPV-2	CCoV	N°(+)
C1.	+	+	+	20 (4.0%)
C2.	+	+		248 (49.4%)
C3.		+	+	3 (0.6%)
C4.	+		+	5 (1.0%)
C5.	+			63 (12.5%)
C6.		+		122 (24.3%)
C7.			+	2 (0.4%)
C8.				39 (7.8%)

Percentage of positives calculated based on the total collected samples.

### 3.2 Phylogenetic analyses

**Figure 1:**
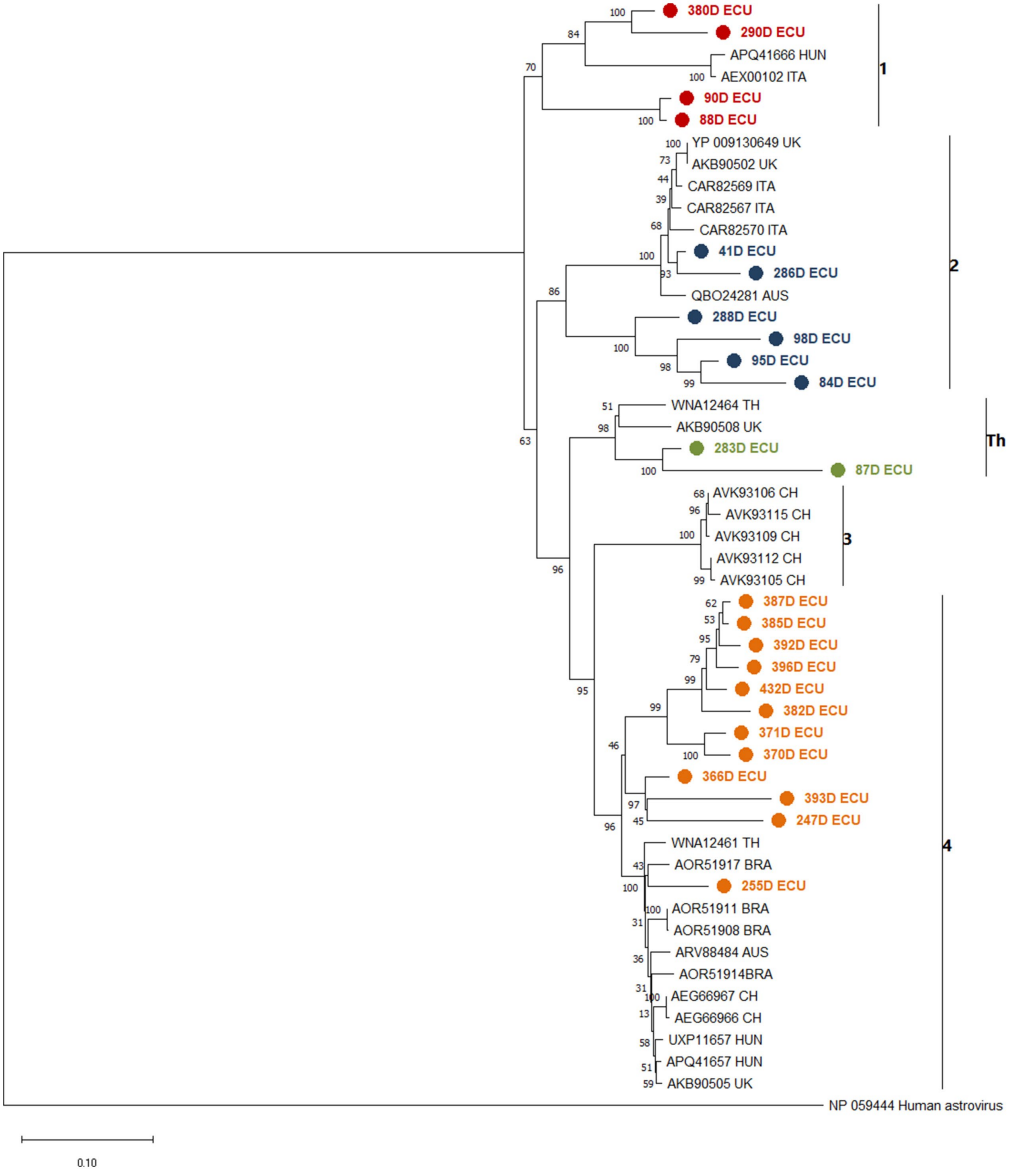
Phylogenetic analysis between CaAstV sequences obtained in this study and translated into amino acids, and other sequences collected from NCBI from Australia (AUS), Brazil (BRA), United Kingdom (UK), Italy (ITA), Hungary (HUN), Thai-land (TH) and China (CH), based on AA of complete ORF2 coding protein due to interest in variation in capside protein formation and changes in capside protein functionality, created by a Jones-Taylor-Thornton (JTT) model, suggested by the MEGA 7 software as the most suitable for the set of sequences, using the boot-strap method with 1,000 replicates. The sequences are clustered and identified against clades 1 to 4 and Th. Sequences obtained near lineage 1 are painted red, lineage 2 is painted blue, lineage 3 is painted green and lineage 4 is painted orange. All sequences obtained in this study were marked with ● in front. The bootstrap percentage is marked in all nodes.

Phylogenetic analysis yielded a tree that divided the sequences retrieved from GenBank into clades according to the 4 previously identified lineages (10), and an additional hypothetical lineage reported in Thailand (12). The samples sequenced in this study were distributed along the lineage clades, with 4 samples close to lineage 1, 6 samples close to lineage 2, two samples close to the Thai lineage and 12 samples close to lineage 4 (Figure 1). When analyzing the nucleotide and amino acid similarity of the sequences obtained in this study with those collected in GenBank, it was found that the sequences close to lineage 4 have a high percentage of similarity (86.9–93.5%), while the sequences close to lineage 1 and Thai lineage show between 76.1 and 84.3%. The sequences obtained close to lineage 2 were divided into two groups, two samples with 94 and 96% similarity to the reference sequences (41D and 286D) and four samples with similarity between 78 and 83% (288D, 98D, 95% and 84D). This indicates a level of genetic variability that surpasses what has been observed in previously identified sequences within each lineage, with a range of 0 to 5% (Supplementary Table 2).

### 3.3 Recombination analysis

**Figure 2:**
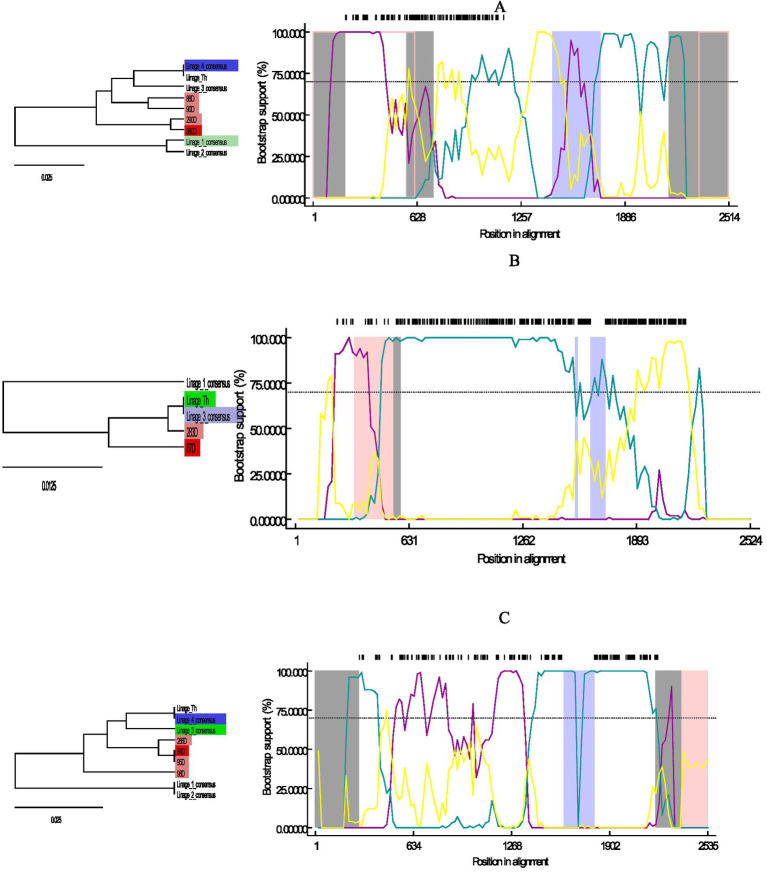
Graph of Bootscan analysis and descriptive tree for the sequences obtained here compared to the consensus sequences of lineages 1, 2, 3, 4, Th; in RDP4 version v.4. **(A)** Analysis for sequences 380D, 290D, 90D, and 88D close to lineage 1. **(B)** Analysis for sequences 84D, 95D, 98D and 288D close to lineage 2. **(C)** Analysis for sequences 87D and 283D close to lineage Th. Trees are color-coded according to their relationship to the recombination event: Red = Possible recombinant, Pink = Sequences with the same recombinant event, Green = Major parent, Blue = Minor parent, Light blue = Sequence used to infer the minor parent, Light green = sequence used to infer the major parent.

The recombination analysis performed on the three lineages of chosen sequences showed possible recombinant events in each of them. Sample 380D, close to lineage 1, showed recombinant events at the beginning and end of its sequence and in two segments within the sequence (Gray area; Figure 2A); where lineage 4 was shown as a possible minor parent, and lineage 1 is used to assume the major parent. Sample 84D, close to lineage 2 showed possible recombinant events at the extremes of the sequence and in the middle (Figure 2B) taking lineages 3 and 4 as the possible parents. Finally, sequence 87D, close to the Th lineage, showed possible recombinant events before 631 bp (Figure 2C), with the Th lineage being its possible major parent, and lineage 3 being used to assume the minor parent. The lack of sufficient parents limits the analyses, which is reflected in the result as sequences are used to assume the major/minor parent.

### 3.4 Capsid protein structure prediction

**Figure 3:**
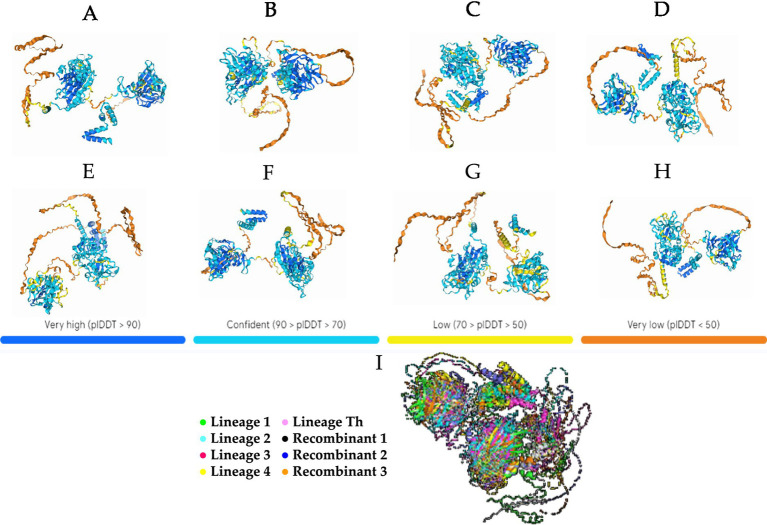
Theoretical 3D model of CaAstV capsid protein folding generated by AlphaFold2 projected onto the predicted local distance difference test (pIDDT) by the program. **(A)** Prediction for a lineage 1 sequence. **(B)** Prediction for a lineage 2 sequence. **(C)** Prediction for a lineage 3 sequence. **(D)** Prediction for a sequence of lineage 4. **(E)** Prediction for a sequence of lineage Th. **(F)** VP protein prediction for the potential 30D recombinant. **(G)** Prediction of the VP protein for the recombinant 84D theoret. **(H)** Prediction of the VP protein for the recombinant 87D theoret. The colors in the 3D protein foldings are based on the confidence level on these or plDTT (predicted local Distance Difference Test): Blue = >90%, Light Blue = between 90 and 70%, Yellow = between 70 and 50%, Orange = <50%. **(I)** 3D protein alignment of the 5 lineages and recombinant theorists in Pymol: Green = Lineage 1, Celeste = Lineage 2, Fuxia = Lineage 3, Yellow = Lineage 4, Pink = Lineage Th, White = Recombinant 1, Blue = Recombinant 2, Orange = Recombinant 3.

The eight 3D proteins generated by the AlphaFold2 program showed generally high plDTT at the two generated centers of the structure and low plDTT at the ends (Figures 3A–I). With similar behavior, the protein structures showed visible similarity at the two centers of the structure formed in all generated proteins, and obvious variability at the end of the structures, which showed differences in each of the generated proteins (Figure 3I).

## 4 Discussion

Viruses represent a primary cause of gastroenteritis in animals, especially in young animals (28), and infections caused by astroviruses are associated with this condition in most species (1). Furthermore, astroviruses have been detected around the world; however, their prevalence and genetic diversity remain largely unexplored. CaAstV has been detected in animals that presented diarrhea in percentages of 2.2–26.9% by molecular techniques in some previous studies (3, 6, 14, 16, 29). The virus was detected by TaqMan RT-qPCR in 66.93% of the samples (336/502). Recent studies detected in 7.5% of the samples studied by conventional RT-PCR and in 15% of the samples with the SYBR-Green RT-qPCR method (6, 14, 16), suggesting that there is a higher detection sensitivity of the latter method (30). The age of detection in this study encompassed animals ranging from two to 180 months old. In addition, it was possible to quantify the amount of viral particles present in the samples. This suggest that CaAstV can be present and is distributed in animals of all age groups, as previous studies have indicated (18, 29, 30).

The fact that the second youngest group of canines that had samples collected, aged 3 to 12 months, showed the highest presence of CaAstV may be attributed to the loss of the innate immunity granted by the mother during lactation (31), characteristic of the first moments of life. When this protection is lost, the canines exhibit an increased susceptibility to a more virulent infection, leading to a greater severity manifestation of the disease (6, 19). The presence of positive samples in the youngest canines, from 0 to 2 months, represents a risk for the health of these animals, since previous studies have mentioned an increase in the severity of the disease in canines up to 7 days of age (4, 19, 28). On the other hand, canines from 13 to 48 months, which refers to young adults, the low number of positive samples in this group may be due to the fact that they already have antibodies for CaAstV and are able to confront the virus, or due to their advanced immune system, the infection does not spread in them (13, 29). Despite the above, all age groups analyzed showed similar viral loads on average (Table 3); although the virulence of CaAstV appears to show no difference depending on the age of the infected canine, further studies are needed to identify the viral behavior under infection and its pathogenicity in depth, as risk factors that increase susceptibility to the disease are common.

Co-infections of other viruses with CaAstV have previously been reported as a possible cause of more dangerous gastrointestinal diseases (20). Co-infection with CPV-2 (Table 4) was more common than single CaAstV infections; it has been documented that simultaneous infections with these two viruses can cause hyperhemorrhagic diarrhea, mainly in cases of co-infections with CPV-2 variants 2b and 2c (6). Moreover, three samples were found to be co-infected with CCoV, although this has been previously reported in symptomatic dogs, no additional pathogenic effect has been highlighted (30, 32); however, given the easiness of transmission of CCoV via the respiratory route, further studies are needed. Finally, other studies have identified co-infections with other enteric viruses such as Kobuvirus, Sapovirus, Vesivirus, Circovirus, Papillomavirus and Calicivirus, associating them with gastrointestinal diseases with more severe symptoms and consequently higher mortality (13, 16, 19, 20, 33), additional studies are essential to determine the presence and potential co-infections with these pathogens.

The ORF2-based phylogenetic tree revealed the presence of 4 distinct CaAstV lineages within the analyzed samples (Figure 1), including the single lineage previously reported in Thailand (10, 12). Given that these lineages correspond to sequences reported in several regions of Europe, Asia and North and South America, the appearance of these lineages in Ecuador implies that there are unidentified epidemiological events where this virus has entered the country. There are no reports of CaAstV in bordering countries, only Uruguay and Brazil are the single countries in South America with reports of this virus (17, 18); more extensive regional studies are needed to identify the behavior of the virus in closer localities within the region. The nucleotide and amino acid sequences showed variability in comparison with the sequences deposited in GenBank since ORF2 is the hypervariable region of the virus that encodes the capsid, and sequences such as 255D ECU (Table 5), maintain a NT similarity rate greater than 90% with sequences from nearby regions such as Brazil, and from more distant regions such as Australia (34, 35). However, some of the sequences showed NT and AA similarity percentages between 70 and 80%, comparable to the variability between lineages, which raises the possibility of active mutagenic or recombinant events in the CaAstV strains circulating in the country. Several of these possible events have been previously reported in China and Vietnam. Furthermore, Astroviruses have been recognized as susceptible to these occurrences, suggesting that similar events may be present in the strains examined in this study (7, 9, 12, 21, 32).

Two of the possible recombinant events analyzed were shown at the beginning and at the end of the ORF2 sequence; it has been previously reported that these areas show the highest rate of genetic variability in *Mamastrovirus*, so these mutations in specific regions could have promoted the appearance of recombination events (9, 21, 36, 37). The appearance of these strains may be related to more severe effects of enteric disease caused by them, consequently it is necessary to analyze *in vitro* infections by these strains, as well as isolate them by lineage. In the analysis of possible recombinant strains 1 and 3, one of the parents was classified as sequence used to infer the major/minor parent, indicating that there is insufficient information on the parents and there are missing sequences between the appearance of the possible recombinant and the previously reported lineages (25, 38). It is possible that there are infections of more than one lineage at the same time in CaAstV-infected canines, this could be promoting the recombination of these sequences; however, in order to identify these events, it is necessary to perform Next-generation sequencing (NGS) to identify the diversity of viral genomes that exist in infected canines (19, 39). More epidemiological and molecular studies are needed to enrich the existing information and fill the gaps in the genetic behavior of CaAstV.

The 3D proteins constructed using AlphaFold2 showed highly similar structures between them, being more similar and reliable according to the program at its center, while demonstrating greater variability and reduced reliability at the extremes (40, 41). The similarity in structures may indicate that the virus maintains a similar and functional structure despite changes in lineages and possible recombinant strains (10, 12, 27, 40). The unreliability in the folding of the protein at the ends may be due to the fact that the behavior and function of these parts is based on the binding of protein units for the formation of the viral capsid (42). Since the structures are not identical, these alterations in protein folding may be influencing an increased pathogenicity of the virus as has been observed in other *Mamastrovirus* (43–45). However, since these data are not *in silico* assays, it is not possible to be certain that the three-dimensional shape of the protein behaves in this way until other methods, such as protein crystallization, are applied (46, 47). However, the interaction between the host and peptide regions remains inadequately characterized, thus the prediction of these structures serves as an initial step toward comprehending the relationship between this virus and its host.

## 5 Conclusion

Canine astrovirus is an emerged viral agent associated with gastroenteritis in dogs of diverse ages. Molecular diagnosis indicate higher rates of CaAstV in sick dogs in Ecuador compared to what has been documented in prior studies. The virus can infect animals across all age groups, though younger animals, especially those losing maternal immunity, are at greater risk of severe disease. Co-infections with other viruses like CPV-2 exacerbate gastrointestinal symptoms, leading to more severe outcomes. The genetic diversity of CaAstV, particularly in the ORF2 region, suggests ongoing mutation and possible recombination events, which may influence the virus’s pathogenicity. The presence of various CaAstV lineages in Ecuador, including previously unreported ones, indicates unidentified epidemiological routes of transmission. Further molecular and epidemiological studies are crucial to understand the full genetic behavior of CaAstV, including the role of possible recombinant strains. Structural analysis of viral proteins suggests that while the core structure remains stable, variations at the ends of VP protein may contribute to increased pathogenicity, though additional *in vitro* studies are required to confirm these findings.

## Data Availability

The datasets presented in this study can be found in online repositories. The names of the repository/repositories and accession number(s) can be found in the article/Supplementary material.
